# Editorial: Targeting Dysregulated Inflammation to Treat Cardiovascular Diseases

**DOI:** 10.3389/fcell.2022.926086

**Published:** 2022-05-16

**Authors:** Mabel Buelna-Chontal, Shyam S. Bansal, Jonatan Barrera-Chimal, Luca Liberale

**Affiliations:** ^1^ Department of Cardiovascular Biomedicine, National Institute of Cardiology, I.Ch., Mexico City, Mexico; ^2^ Department of Physiology and Cell Biology, The Dorothy M. Davis Heart and Lung Research Institute, The Ohio State University, Columbus, OH, United States; ^3^ Centre de Recherche de l'Hôpital Maisonneuve-Rosemont, Faculté de Médecine, Centre affilié à l'Université de Montréal, Montréal, QC, Canada; ^4^ Department of Internal Medicine, University of Genoa, Genoa, Italy; ^5^ IRCCS Ospedale Policlinico San Martino Genova–Italian Cardiovascular Network, Genoa, Italy

**Keywords:** inflammation, cardiovascular disease, inflammaging, atherosclerosis, aging, monocytes, microvescicles, COVID-19

When tightly regulated, inflammation plays important roles in the human physiology ranging from host-defense to tissue-repair and -regeneration, but also in the adaptation to stress and restoration of homeostasis. On the other hand, dysregulated inflammatory cascades have also been linked to accelerated aging, metabolic shifts and a plethora of degenerative diseases including cardiovascular. Specifically, sustained low-grade sterile chronic inflammation associated either with aging (inflamm-aging) or cellular metabolic stress in response to chronic nutrient excess has long been identified as a major player in atherosclerosis and its acute ischemic complications such as myocardial infarction and ischemic stroke (Liberale et al., 2020; Liberale et al., 2022). In most cases inflammatory mediators act in concert with other known regulators of atherosclerosis including modified lipoproteins and reactive oxygen species, all acting on central regulators of inflammation such as the NLRP3 inflammasome and the transcription factor NF-кB, to promote the synthesis and release of cytokines (Liberale et al., 2021a). Despite the high amount of experimental literature that inspired the so-called “inflammatory theory of atherosclerosis”, its bench to bedside translation required many years to come mainly due to i) the complexity of inflammatory processes facilitated by different mediators interconnected at different levels and often regulating each other, and ii) the high likelihood of side-effects of the interventions aimed at modifying immunological functions. Only very recently and after a series of futile trials that the role of anti-inflammatory molecules in secondary cardiovascular prevention was proven in the clinics also (Ridker et al., 2017; Tardif et al., 2019; Liberale et al., 2021b).

With the aim of improving the effectiveness of current therapeutic strategies to treat cardiovascular diseases and reduce unwanted side-effects, understanding the whole cascade of atherosclerosis which encompasses dysregulated inflammation is an important need of today’s cardiovascular research. In this context, the current Research Topic for *Frontiers in Cell and Developmental Biology* aims to collect novel evidence in the field and to help integrate the available knowledge with the final aim to reduce the global cardiovascular disease burden.

In their article *Leucine-Rich α-2-Glycoprotein 1 Suppresses Endothelial Cell Activation Through ADAM10-Mediated Shedding of TNF-α Receptor* Pang et al. report for the first time the immunomodulatory role of LRG1 through the shedding of TNFR1, a known activator of the inflammatory cascade in the endothelial cells. Based on the above, the authors suggest LRG1 as a novel therapeutic target in inflammatory and autoimmune diseases.

Then, Chiva-Blanch et al. from Institut de Recerca Hospital Santa Creu i Sant Pau-IIB Sant Pau in Barcelona (Spain) report on successful aging, showing that such a desirable feature associates with low levels of circulating micro-vesicles released by the endothelial cells and the platelets, and is an indication of their low pro-inflammatory status. Their report *Functional and Cognitive Decline is Associated With Increased Endothelial Cell Inflammation and Platelet Activation: Liquid Biopsy of Microvesicles in Community- Dwelling Octogenarians* further builds on the evidence of inflammaging as a mediator of cardio- and cerebrovascular diseases.

From Catholic University of the Sacred Heart in Rome (Italy), Vinci et al. describe the heterogeneity of circulating monocyte subsets in patients with non-ST-elevation acute coronary syndrome showing downregulation of patrolling cells and prevalence of pro-inflammatory features in case of plaque rupture. Of much interest in their work *A Novel Monocyte Subset as a Unique Signature of Atherosclerotic Plaque Rupture* they report the association of a novel monocyte population named pre-classical monocytes with atherosclerotic plaque rupture and macrophage infiltration. Also, they state that the clinical outcome depends on the individual response to inflammatory insults.

In their contribution *Neutrophil Migratory Patterns: Implications for Cardiovascular Disease*, Dahdah et al. from The Ohio State University (US) review the mechanisms involved in leukocyte margination and demargination. Specifically, they discussed the role of neutrophil migratory patterns during diabetes and cardiovascular diseases, concluding that more work is needed to fully understand whether margination or demargination have positive or negative effects on their pathophysiology, and whether the molecules regulating such mechanisms may potentially become future therapeutic targets.

With a very timely article, Martinez-Salazar et al. review the effect of Severe Acute Respiratory Syndrome Coronavirus 2 (SARS-CoV-2) on the vasculature. In their comprehensive article *COVID-19 and the Vasculature: Current Aspects and Long-Term Consequences* the authors reports on what is SARS-CoV-2 and how it enters and replicates in our bodies. Then, they summarize what we know about the effects of this virus in the vasculature and the development of CV diseases with a final hint on long COVID-19 syndrome and its potential impact on cardiovascular health.

*A Systematic Review of the Mechanisms Involved in Immune Checkpoint Inhibitors Cardiotoxicity and Challenges to Improve Clinical Safety* is the article written by Rubio-Infante et al. from Monterrey Institute of Technology and Higher Education (Mexico). After analyzing the potential immune mechanisms underlying cardiac adverse events of anti-cancer agents, they propose that self-antigens released from cardiac tissues or cancer cells, and the severity/advancement of cancer play important roles in determining the cardiotoxicity potential of immune checkpoint inhibitors.

In a last piece *Modern concepts in cardiovascular disease: inflamm-aging*, Puspitasari et al. from the Center for Molecular Cardiology of the University of Zurich (Switzerland) explore the role of the chronic low-grade inflammation that develops with age in the pathophysiology of atherosclerosis and acute ischemic cardio-and cerebrovascular afflictions. After reviewing the molecular pathways underlying inflamm-aging, the authors also explore the potential of anti-inflammatory approaches as cardiovascular drugs.

Editors and authors hope that the present issue provides new evidence as well as an updated summary on the intriguing and always evolving field of dysregulated inflammation in cardiovascular diseases. In consideration of the recent successful clinical trials, the bench-to-bedside transition of such concepts is feasible and has already shown significant therapeutic potential. We envisage that the approaches aimed at regulating inflammation to be a future of clinically viable therapies that will facilitate personalized medicine in cardiology.
